# Association of HLA-DRB1 and -DQB1alleles and haplotypes with rheumatoid arthritis in a Pakistani population

**DOI:** 10.1186/ar4275

**Published:** 2013-08-22

**Authors:** Ambreen Gul Muazzam, Atika Mansoor, Lubna Ali, Saima Siddiqi, Abdul Hameed, Muhammad Ajmal, Kehkashan Mazhar

**Affiliations:** 1 Institute of Biomedical and Genetic Engineering, 25 Mauve Area, G9/1, Islamabad 44000, Pakistan

**Keywords:** MHC class II, complex disease, genetic association, rheumatoid arthritis, Pakistani population, meta-analysis

## Abstract

**Introduction:**

Rheumatoid arthritis is an autoimmune disease with poorly understood pathophysiology. Genetic components of disease etiology, especially human leukocyte antigen (HLA) associations, are well known. Ethnic differences account for a number of variations in disease association with the HLA locus and there seem to be differences in various studies regarding its genetic predisposition. This study was aimed at determining the contribution of DRB1 and DQB1 components of HLA class II in rheumatoid arthritis in a Pakistani cohort.

**Method:**

For this study, 110 patients and 120 healthy controls from the same geographical area and matched ethnicity were enrolled. Blood DNA was isolated from all the subjects and HLA alleles were typed following allele specific amplification. Subsequently, haplotypes were generated and allelic and haplotype distribution frequencies were compared among the patients and controls using χ^2 ^and Arlequin software. The data obtained by this analysis were also compared with other reported associations found in the Pakistani population by meta-analysis.

**Results:**

HLA allelic status was determined among the patients and controls from the same geographical area to account for differences in ethnicity and environmental factors. Significant associations were found for alleles as well as haplotypes among the patients of rheumatoid arthritis. DRB1*10, DQB1*05 and DQB1*602 were found to be associated with disease susceptibility, whereas DRB1*11 and DQB1*02 had protective effect against the disease. Similarly, haplotype DRB1*10-DQB1*05 was associated disease risk, whereas DRB1*07-DQB1*02 and DRB1*11-DQB1*0301 had a protective effect.

**Conclusion:**

There is a significant DRB1and DQB1 allele and haplotype association with rheumatoid arthritis susceptibility and protection.

## Introduction

Rheumatoid arthritis (RA) is a complex, long-term disease causing inflammation of joints and surrounding tissue. Inflammation is misdirected to attack one's own joints, an autoimmune disease caused by the over expression of immune response which causes synovitis. Long-term disease can lead to major functional disability; therefore, early diagnosis and determination of risk factors are important in effective disease management. RA usually requires lifelong treatment, including medications, physical therapy, exercise, education and possible surgery [1]. Since early treatment for RA can delay joint destruction, assessment of predisposition to the disease and determination of risk factors become all the more important for effective disease management.

It is difficult to determine the prevalence of RA due to the heterogeneity of disease presentation but it is estimated that about 1% of people are affected with RA worldwide. There are also some ethnic differences [2]; for example, rheumatoid arthritis affects about five to six percent of some Native American groups, while the rate is very low in some Caribbean peoples of African descent, and South East Asian populations. Similarly, women get the disease more often than men [3].

The genetic factor of the disease is also well established based on the fact that the risk rate is about two to three percent in people who have a close relative with rheumatoid arthritis, such as a parent, brother or sister [4]. It is also well known that genetic factors account for 60% of the disease risk. Various genetic factors have been reported to be positively or negatively associated with the disease. Recent genome wide association studies have added few new loci, increasing the susceptibility loci to about 30 [5]. The role of the major histocompatibility complex (MHC) genes account for 50% of the genetic susceptibility in most autoimmune diseases, in RA the human leukocyte antigen (HLA) region contributes most to the genetic risk. Specifically, there have been reports on the association of class II antigens DRB1. It has been reported that DRB1* 0101, *0401, *0404, *1001 and *1402 play a key role in the predisposition to the most severe form of the disease [1,6-8]. The presence of two alleles with shared epitopes in patients, that is, the conserved epitope amino acid sequence Q/R(K/R)RAA numbers 70 to 74 in the third hypervariable region of the DRß1 chain, poses a higher risk and greater disease intensity. Some of the other DRB1 alleles, for example, HLA-DRB1*0103, *0402, *12, *1301, *1302 and *1304 carrying D/QERAA sequence of amino acids in the third hyper-variable region are considered protective [5,9].

It is worth mentioning that the HLA-DRB1 locus reported as the largest predisposing genetic risk factor to RA among Caucasian and Asian populations also show considerable variation among different ethnic groups. For example, HLA DRB 04 is reported to be associated with the disease in Caucasian and Asian populations whereas DRB 01 is associated with the Israeli Jewish population [4]. These findings point towards genetic heterogeneity in RA susceptibility across different ethnic groups.

The Pakistani population has a varied ethnic background with a long history of invaders in this region. The current population consists of distinct ethnic groups confined to different regions of the country with some admixture. These populations include Pathan, Hazara, Kalash, Burusho, Kashmiri and Punjabi populations from the northern part of the country, and Baloch, Brahui, Sindhi, Makrani, Parsi and Mohanna populations from the southern part of the country. Recent molecular genetic studies have shown all ethnic groups to cluster together and, in general, show a close relatedness with the European and Middle-Eastern populations [10-12].

This current study was undertaken in order to determine the genetic risk associated with HLA class II alleles in our population cohort reported already in various other population studies. Therefore, HLA class II DRB1 and DQB1 allelic associations were studied. Haplotype associations for the two loci were also studied in order to determine any possible synergistic effect of the associated alleles.

## Material and methods

### Subjects

A total of 110 RA patients from the Out-Patient Department, Rheumatology Clinic of the Pakistan Institute of Medical Sciences, Islamabad, Pakistan were selected. Whereas, 120 age- and ethnicity-matched, random healthy controls with no apparent disease were enrolled in the study from the same geographical area. The samples collected were from the northern area including Pathan, Punjabi and Kashmiri. The study was conducted following ethical guide lines of the 2000 Helsinki Declaration and duly approved by the Institute of Biomedicl and Generic Engineering (IBGE) ethical committee. Blood samples (5.0 ml) were collected after written informed consent was obtained from all participants for their enrollment.

### RA patients

The patients met the following criteria: patients presented adult onset of the disease, that is, at 16 years of age or later. Only patients fulfilling the American College of Rheumatology criteria were considered RA patients [13]. Data consisting of their age, gender, ethnicity, age at disease onset, RF (rheumatoid factor) status and ESR (erythrocyte sedimentation rate) were generated by well trained personnel.

### HLA typing

DNA was isolated from the blood of all patients and control samples. PCR analyses for HLA class II were carried out following the method of Bunce *et al. *[14] and allelic status for DRB1 and DQB1 loci was subsequently determined.

### Statistical analysis

Allelic distribution at two loci in patients and controls was analyzed by using χ^2 ^or Fisher's exact test. Odds Ratio (OR) and Confidence Interval (CI) were calculated using VassarStats (Richard Lowry, Poughkeepsie, NY USA) [15]. Two locus haplotypes were generated by Arlequin (v3.11) software (Laurent Excoffier 1998-2007, Computational and Molecular Genetics Lab (CMPG), Zoological Institute, University of Bern, Germany) using the allele status at two loci. Bonferroni correction was applied to the *P-*values [16]. From the number of patients and the number of controls reported in previous studies, OR and CI were calculated using an online calculator [17]. Allele frequencies, OR and CI of all the studies carried out up until April 2013 on the Pakistani population [18-20] and the present study were compared, the data were analyzed using Comprehensive Meta-Analysis (ver 2.0) software (Biostat Inc. Englewood, NJ, USA).

## Results

The patients' clinical profiles are shown in Table 1. Allelic and haplotype distribution among the patients (110) and controls (120) are given for the two loci, that is, DRB1 and DQB1 in Tables 2 and 3. Significant associations were obtained at both loci.

**Table 1:** Demographic and clinical characteristics of patients with rheumatoid arthritis.

	Female	Male	Population
**Number (%)**	98 (87.5)	14 (12.5)	112 (100)
**Age (Mean ± SD, years)**	41.22 (11.24)	40.57 (14.7)	41.14 (11.65)
**Duration of illness (Mean ± SD, years)**	9.70 (8.06)	6.86 (3.9)	9.35 (7.71)
**Signs and symptoms:**	**No. (%)**	**No. (%)**	**No. (%)**
**Pain**	5 (5.10)	4 (28.57)	9 (8.04)
**pain + swelling**	6 (6.12)	2 (14.29)	8 (7.14)
**pain + stiffness**	10 (10.20)	0 (0)	10 (8.93)
**pain + swelling + stiffness**	77 (78.57)	7 (50.0)	84 (75.0)
**not known**	0 (0)	1 (7.14)	1 (0.89)
**RF positive**	57 (58.16)	6 (42.86)	63 (56.25)
**Positive family history**	43 (43.88)	2 (14.29)	45 (40.18)

**Table 2:** Allelic distribution of patients and controls at DRB1 and DQB1 loci.

n (valid%)			n (valid%)		
DRB*1	Patients	Controls	DQB*1	Patients	Controls
01	8 (3.7)	9 (3.8)	**02**	**35 (15.9)**	**76 (33)**
03	30 (13.8)	50 (21.4)	04	6 (2.7)	6 (2.6)
04	21 (9.6)	32 (13.7)	**05**	**81 (36.8)**	**27 (11.7)**
07	33 (15.1)	32 (13.7)	0301	37 (16.8)	51 (22.2)
08	1 (0.5)	2 (9)	0601	37 (16.8)	47 (20.4)
09	3 (1.4)	0 (0)	**0602**	**13 (5.9)**	**1 (0.4)**
**10**	**44 (20.2)**	**14 (6)**	0603	8 (3.6)	16 (7)
**11**	**7 (3.2)**	**34 (14.5)**	0604-09	3 (1.4)	6 (2.6)
12	3 (1.4)	4 (1.7)			
13	9 (4.1)	15 (6.4)			
14	3(1.4)	2 (0.9)			
15	55 (25.2)	40 (17.1)			
16	1 (0.5)	0 (0)			

Total (2n)	218 (100)	234 (100)	Total (2n)	220 (100)	230 (100)

Missing DRB1: patients = 2, controls= 0

Missing DQB1: patients = 0, controls = 4

**Table 3:** Top 10 major haplotypes and their frequency distribution among patients and controls.

Haplotype DRB1-DQB1	n (%) in groups	
	
	Patients (2*n *= 220)	Control (2*n *= 230)
DRB1*03-DQB1*02	15 (6.8)	25 (10.8)
DRB1*07- DQB1*02	4 (1.8)	26 (11.3)
DRB1*10- DQB1*05	30 (13.6)	6 (2.6)
DRB1*11- DQB1*0301	9 (4.0)	25 (10.8)
DRB1*15- DQB1*0601	32 (14.5)	28 (12.1)
DRB1*13- DQB1*0603	2 (0.9)	7 (3.1)
DRB1*01- DQB1*05	4 (1.8)	7 (3.1)
DRB1*04- DQB1*02	1 (0.45)	7 (3.1)
DRB1*01- DQB1*05	0 (0)	7 (3.1)
DRB1*04- DQB1*0601	6 (2.7)	1 (0.45)

### Allelic distribution

It is evident from Table 1 that at the DRB1 locus *1501 is the most prevalent (25.2%) allele among the patients followed by *10 (20.2%) and *07 (15.1%). In the control population the most prevalent allele is *03 (21.4%) followed by *15 (17.1%) and *11 (14.5%).

At the DQB1 locus *05 was the most prevalent allele (36.8%) followed by *0601 and *0301 with equal valid frequency of 16.8% among the patients, whereas in the controls *02 was most prevalent (33%), followed by 0301 (22.2%) and 0601 (20.4%).

### Allelic association

Allelic frequencies were compared between the patients and the control groups using χ^2 ^or Fisher's exact test. At the DRB1 locus it was found that the frequencies varied significantly between the two groups for the DRB1*10 as well as the DRB1*11 loci (Table 4). DRB1*10 was found at a higher frequency (20.2%) among the patients, whereas its frequency among the control group was quite low (6.0%) with *P-*value <0.0001 and corrected *P *= 0.0013, OR 3.97 (95% CI = 2.1 to 7.4). This significance was retained even after Bonferroni correction and, therefore, shows an association with RA. The frequency of DRB1*11 was quite low in the patient group (3.2%) and relatively higher among the control group (14.5%) with *P*-value <0.0001, corrected *P *= 0.0013, OR 0.19 (95% CI = 0.08 to 0.45). DRB1*11, therefore, also showed a significant difference between the two groups.

**Table 4:** Statistical analysis of patients and controls at DRB1 locus.

Alleles in (DRB)	*P*-value	odds ratio	CI	*P-c*orrected
	0.92	0.95	0.36 to 2.5	11.96
03	0.03	0.59	0.35 to 0.96	0.44
04	0.18	0.67	0.37 to 1.2	2.37
07	0.65	1.13	0.66 to 1.9	8.45
08	1	0.5346	0.04 to 5.9	13
**10**	<.0001	3.97	2.1 to 7.4	0.0013
**11**	<.0001	0.19	0.08 to 0.45	0.0013
12	1	0.8	0.17 to 3.6	13
13	0.28	0.63	0.26 to 1.47	3.627
14	1	1.6	0.26 to 9.7	13
15	0.03	1.63	1.03 to 2.58	0.43

*P *<0.05 is considered significant

Allelic frequencies at the DQB1 locus were also similarly compared between the two groups. It was found that at the DQB1 locus allelic distribution varied significantly for three alleles *viz *DQB1*02, DQB1*05 and DQB1*602 (Table 5). Frequency of DQB1*02 was significantly high in the control group with *P*-value ≤0.0001; corrected *P *= 0.0008; OR = 0.38 (CI = 0.24 to 0.6 at 95% level). Allelic frequency also varied significantly at DQB1*05, being higher among the patients with *P-*value ≤0.0001, corrected *P *= 0.0008, OR = 4.38 (CI = 2.69 to 7.1) at 95% level). Similarly, the frequency was significantly high in patients for the allele *602 with *P *= 0.0008, corrected *P *= 0.006, OR 14.38 (CI = 1.8 to 110 at 95% level).

**Table 5:** Statistical analysis of patients and controls at DQB1 locus.

DQB	*P*-value Pearson chi	OR	CI	Corrected *P*
**02**	≤0.0001	0.383	0.24 to 0.60	0.0008
04	0.92	1.04	0.33 to 3.29	7.36
**05**	≤0.0001	4.38	2.69 to 7.1	0.0008
0301	0.15	0.709	0.44 to 1.13	1.21
0601	0.32	0.787	0.48 to 1.2	2.59
**0602**	0.0008	14.38	1.8 to 11.0	0.006
0603	0.117	0.505	0.21 to 1.2	0.934
0604-09		0.516	0.12 to 2.08	

*P *<0.05 is considered significant.

Significant associations obtained were also compared with three other studies published on the Pakistani population for the DR locus, namely Hameed *et al*. 1997, Ali *et al*. 2006 and Naqi *et al*. 2011 [18-20]. At the DQ locus the results were compared with one other study on the Pakistani population, Ali *et al*. 2006 [20].

**Figure 1:**
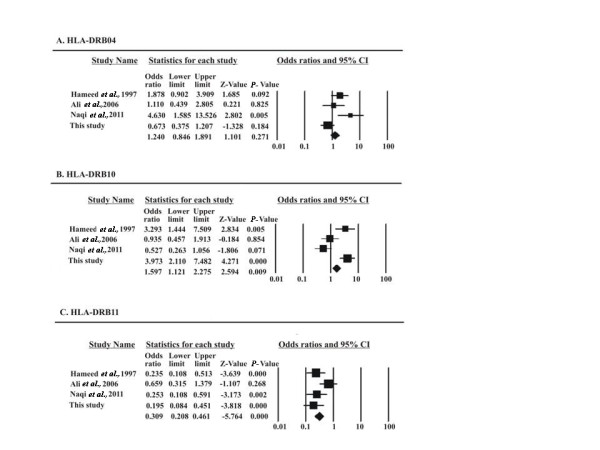
**Forest plots for all DRB significant association studies in the Pakistani population**. Plots show the effect size and precision for each study and the combined effect of all studies. Filled boxes show proportional size of the study weight.

Meta-analysis was performed for all the significant allelic associations reported in these studies. Forest plots show the statistical results of all the analyses. At the DRB locus (Figure 1) there are three associations reported so far, DRB 04 and DRB 10 as disease associated and DRB 11 as protective. As shown in Figure 1A, the *P-*value for cumulative effect of DRB 04 is >0.05 (*P *= 0.271); therefore, we can conclude that the DR 04 effect is not significant in this population. For DRB 10 (Figure 1B) there seems to be a disease association effect with *P-*value = 0.009, and DRB 11 (Figure 1C) is protective with cumulative *P *<0.001.

**Figure 2:**
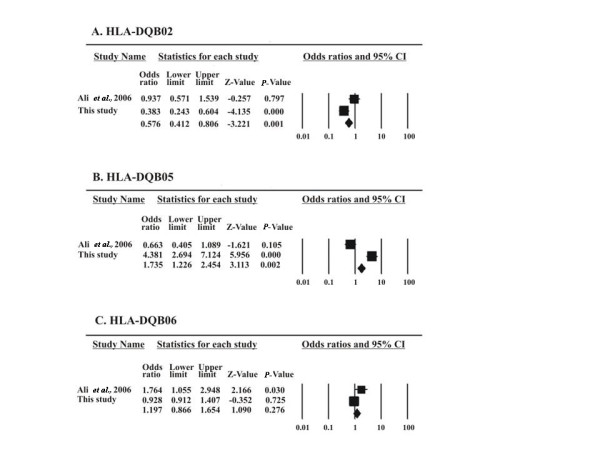
**Forest plots for all DQB significant association studies in the Pakistani population**. Plots show the effect size and precision for each study and the combined effect of all studies. Filled boxes show proportional size of the study weight.

At the DQ locus we obtained three significant alleles and all three were compared with the only published data at this locus: that is, Ali *et al*. 2006 [20]. The cumulative protective effect of DQB 02 was significant with *P *= 0.001 (Figure 2A), for DQB 05 also there was significance with *P *= 0.002 towards the protective effect (Figure 2B), whereas the protective effect of DQB 06 was not significant (Figure 2C).

### Haplotype distribution

In addition to the allele frequencies, two locus haplotype frequencies were also calculated from all possible haplotype combinations generated (patients = 52, controls = 59) by Arlequin software. All major haplotypes obtained in the two groups are shown in Table 5; minor haplotypes with less than 1% presentation are not included. Among the patients, DRB1*15- DQB1*0601 was the most common haplotype found (14.5%), and DRB1*10- DQB1*5 was the second most frequent haplotype (13.6) observed. Among the control group DRB1*15- DQB1*0601 was the most common (12.1%) and DRB1*07- DQB1*02 was the second most frequent haplotype (11.3%) observed.

### Haplotype association

Comparison of the patients and control group was carried out for the two locus haplotype distribution (Table 6). A significant difference in frequency distribution was observed for three haplotypes *viz *DRB1*07-DQB1*02 (*P *<0.0001; and corrected *P *= 0.005, odds ratio = 0.105, CI = 0.03 to 0.33), DRB1*11-DQB1*0301(*P *<= 0.00564; corrected *P *= 0.029, odds ratio = 0.28, CI = 0.11 to 0.68) and DRB1*10- DQB1*05 (*P *<0.0001; and corrected *P *= .005 for odds ratio = 12, CI = 4.39 to 32.9). Therefore, DRB1*07-DQB1*02 and DRB1*11-DQB1*0301are protective, whereas, DRB1*10- DQB1*05 is disease-associated. Linkage disequilibrium between DRB1*07 and DQB1*02 was calculated in order to determine the level of association between these two loci (Δ = 0.03, *P *= 0) and similarly between DRB1*11 and DQB1*0301 (Δ = 0.0568, *P *<0.001), and between DQB1*10 and DQB1*5 loci (Δ = 0.049, *P *= 0).

**Table 6:** Statistical analysis of major haplotype associations with patients and controls.

Haplotype DRB1-DQB1	*P*-value	Odds ratio	CI	Corrected *P*
DRB1*03- DQB1*02	0.069	0.5514	0.24 to 1.2	
DRB1*07- DQB1*02	<0.0001	0.1058	0.03 to 0.33	0.005
DRB1*10- DQB1*05	<0.0001	12	4.39 to 32.9	0.005
DRB1*11- DQB1*0301	0.000564	0.2847	0.11 to 0.68	0.029
DRB1*15- DQB1*0601	0.554	1.77	0.83 to 3.77	28

*P *<0.05 is considered significant.

## Discussion

Our study was aimed at determining the effect of HLA class II alleles in rheumatoid arthritis in patients from Pakistan. A cross-sectional case control study was carried out to compare RA patients with a control population. The HLA profile of the control group was in agreement with previous studies [21-23]. Samples collected were not analyzed on the basis of ethnicity as the patients were a mixed population from the northern region who visited the public sector hospital for consultation. Random selection of patients showed a predominance of females with the disease. Various studies, including one from Pakistan, have shown a disproportionate effect of autoimmune diseases, including RA, on middle-aged women [24,25]. The high female ratio is probably due to the involvement of X-linked and hormonal factors that interplay with a number of autoimmune diseases.

The study reports RA disease association with HLA allelic and haplotype variations. Since the Pakistani population shows close relatedness with Caucasian and Middle-Eastern populations [10] we expected similar allelic and haplotype association with the disease as reported for other Semitic races. Surprisingly, our results show some novel associations as well as previously reported ones. In the study we found a significant effect of allelic predisposition for the disease as well as variations that seem to have a protective effect against the disease. There has lately been more focus on the DRB1 locus probably because of the shared epitope hypothesis but we clearly found an effective role of the DQB1 locus as well.

It was found that the alleles DRB1*10, and DQB1*05 were strongly disease associated, with 44 (20.2%) and 81 (36.8%) patients, respectively. Allele DQB1* 602 was also significantly associated with the disease with 13 (5.9%) patients carrying this allele (Table 2). We analyzed the cohort with RF positive patients (58%) and the effect of DQB 602 was enhanced (*P *= 0.0001). Associations with DRB1 or DRB4 reported in other populations [26] were not found. DRB1*10 allelic association reported in this study is in agreement with another published study on patients from South of Pakistan [19] as well as in some Caucasian populations [27], although we got slightly higher significance of association even after Bonferroni correction. All these alleles at the DRB locus have a so-called common shared epitope [28] and, therefore, may be involved in the same functional pathways. It is also worth mentioning that the allele frequency of DRB1*10 is very low in the general population in Pakistan which has been verified in a number of studies [20,21,25], but its frequency is very high in our patient population, which highlights its significance with RA disease. DRB1* 04 was not significantly different in the patients and controls of our cohort although the frequency was higher in the controls (Table 2). In the study of Hameed *et al. *[19], the frequency of DR04 is higher in the patient group; on sub-typing they found frequency of 402 and 403 associated with protection to be quite low. If we exclude these alleles from our 04 samples it may only tilt the trend toward neutrality rather than towards disease association. Comprehensive meta-analysis was performed in order to analyze the allelic significance shown in various studies reported for Pakistani RA patients. As shown in the forest plot, DRB 10 shows a cumulative disease association (*P *= 0.009) further supporting the results of our study and the involvement of RRRAA motif [27]. The significance of DRB 04 shown in other Pakistani population studies is reduced, thus showing noninvolvement of DRB4 in the disease in this region.

At the DQB locus we found a significant association of the disease with DQB 05. No other data on the Pakistani population were available for DQB association, except Ali *et al. *[20]; therefore, we compared our data with one study only. The allelic association remained significant also for the cumulative effect (*P *= 0.002, Figure 2B), showing the significance in this study. Association with DQB1*05 has also been proposed previously in a model suggesting DQB1 *03 and DQB1*05 as predisposing alleles that are in disequilibrium with DR alleles [29,30]. In addition to the allelic association, haplotype DRB1*10-DQB1*05 was highly significant, with 30 patients (13.6%) vs. 6 controls (2.6%) carrying this haplotype. This significance might be due to linkage disequilibrium between DR10 and DQ5. Since both alleles at their respective loci were strongly disease associated, they also appeared in the associated haplotype but it must also be noticed that the haplotype effect gives a higher odds ratio (Tables 4, 5, 6). DQB 602, also significantly disease-associated in our data, showed enhanced disease association with RF positivity. In meta-analysis results we pooled our data for all DQB 06 sub-alleles to compare them with Ali *et al. *[20], who did not subtype for DQB 0602. The significance was lost, showing the effect of 0602 only. It is worth mentioning that in the western population DQB 601 and DQB 602 are both linked to DRB 15 and show a protective effect [31]. In our samples, 602 is not linked with DR15 or any other DRB locus, appears with varied DR loci and shows disease susceptibility.

Since all the results for DQB locus meta-analysis have been compared with only one available study reported so far and our results, although significant, are not in agreement with that previous study, any conclusion drawn for this locus should be taken with due caution.

DRB1*11 was protective with 34 control samples (14.5%) carrying this allele, whereas DQB1*02 was highly protective with 76 control samples (33%) carrying this allele. The protective effect of allele DQB1*02 is reported for the first time in this study although it was reported in haplotype HLA-DQA1*05-DQB1*02 [32]. The effect of the DQ locus (*P *= 0.0008) is more pronounced than the effect of the DR locus (*P *= 0013). Surprisingly, the two loci do not form a valid haplotype, although the DQ locus has a significant presentation in the patients and control population. This could be due to a stronger linkage disequilibrium between DRB1*07 and DQB1*02. It seems that the HLA DRB1*11 and DQB1*02 alleles assort independently. Meta-analysis results with the other three studies on the Pakistani population retained the significance of protective effect of DR 11 (Figure 1C) as well as DQ02 (Figure 2A). DRB1*11 protection is explained due to its DERAA motif at positions 70 to 74 [33].

The haplotype DRB07-DQB02 showed a protective advantage with 26 control samples (11.3%) carrying this combination. Allele DRB1*07 is a common allele in both populations. It is interesting to note that the DRB1*07 allele was reported to confer protection in the Moroccan population with *P *= 0.03 [34].

In addition, haplotype DRB1*11-DQB1*0301 was also significantly protective with 25 control samples compared to 9 patient samples. Here again DQB1*0301 was a common allele in both populations, it has also appeared in the haplotype formation. When we compare the allelic and haplotype effect of DRB1*11 it can be seen from Tables 3, 4 and 6 that there is no significant combined effect of the two alleles as expected due to non-significance of DQ 0301. It may be that the protective effect of DQB1*0301 is due to linkage disequilibrium with DRB1*11, which was confirmed by the LD test (Δ = 0.056). It may also be mentioned that haplotype protection of DRB1*0403-DQB1*O301 has been reported previously [6]. When the protective effect of DQB1*02 is compared in the allelic and haplotype formation of our cohort, there is no significant difference (Tables 4, 5, 6) and it may be concluded that the apparent haplotype effect is only due to linkage disequilibrium between the two alleles reported in various studies [6,35].

## Conclusions

In conclusion, our results show significant association of rheumatoid arthritis with HLA class II alleles DRB1*10 and DQB1*05 as well as DQB1*602. A significant disease protection was also found with alleles DRB1*11 and DQB1*02 not reported previously. Haplotypes DRB1 *10-DQB1 *05 was disease-associated and two haplotypes DRB1* 07-DQB1*02 and DRB1*11-DQB1*0301 were protective against the disease. Meta-analysis of the Pakistani population further strengthens our results. The findings indicate the importance of the need for conducting disease association studies for specific ethnic groups and populations of patients and determine the ever increasing genetic variation of this disease. It might provide an insight into identifying the important pathways involved and will, therefore, help in prognosis and disease management for patients suffering from rheumatoid arthritis.

## Abbreviations

CI: confidence interval; HLA: human leukocyte antigen; OR: odds ratio; RA: rheumatoid arthritis; RF: rheumatoid factor; SE: shared epitope.

## Competing interests

The authors declare that they have no competing interests.

## Authors' contributions

AGM performed all the experimental work and data analysis. AM read the manuscript and guided the experimental and analytical part of the study. LA and SS performed experimental work and helped in data analysis. AH and MA contributed to sample collection and preparation. KM designed the study, reviewed the data and prepared the manuscript. All authors read and approved the final manuscript.
